# CircUBE2D2 (hsa_circ_0005728) promotes cell proliferation, metastasis and chemoresistance in triple-negative breast cancer by regulating miR-512-3p/CDCA3 axis

**DOI:** 10.1186/s12935-020-01547-7

**Published:** 2020-09-14

**Authors:** Dongwei Dou, Xiaoyang Ren, Mingli Han, Xiaodong Xu, Xin Ge, Yuanting Gu, Xinxing Wang, Song Zhao

**Affiliations:** 1 Department of Breast Surgery, The First Affiliated Hospital of Zhengzhou University, 1 Jianshe East Road, Erqi District, Zhengzhou, 450052 China; 2 Department of Information, The First Affiliated Hospital of Zhengzhou University, Zhengzhou, 450052 China; 3 Department of Thoracic Surgery, The First Affiliated Hospital of Zhengzhou University, 1 Jianshe East Road, Erqi District, Zhengzhou, 450052 China

**Keywords:** Triple-negative breast cancer, circUBE2D2, miR-512-3p, CDCA3

## Abstract

**Background:**

Triple-negative breast cancer (TNBC) is a clinically aggressive subtype of breast cancer with a bad prognosis. Chemotherapy is still the standard of care for TNBC treatment. Circular RNAs (CircRNAs) have been recently discovered to be closely involved in the initiation and development of human cancers. Herein, we focus our attention on the functions and underlying mechanisms of circUBE2D2 in TNBC progression and chemoresistance.

**Methods:**

The expression of circUBE2D2, miR-512-3p, and cell division cycle associated 3 (CDCA3) mRNA were determined by qRT-PCR. CCK-8, colony formation, transwell and flow cytometry assays were performed to detect cell proliferation, migration, invasion and apoptosis. Western blot assay was utilized to measure the protein level of CDCA3. RNA pull-down, luciferase reporter and RIP experiments were employed to examine the possible regulatory mechanism of circUBE2D2.

**Results:**

CircUBE2D2 expression was elevated in TNBC tissues and cells. TNBC patients with high circUBE2D2 expression are inclined to present advanced TNM stage, lymph node metastasis and adverse prognosis. Knockdown of circUBE2D2 repressed cell proliferation, migration and invasion in vitro, and impeded tumor growth in vivo. Moreover, silencing of circUBE2D2 reduced doxorubicin resistance of TNBC cells. In-depth mechanism analysis revealed that circUBE2D2 served as a miRNA sponge to protect CDCA3 from the attack of miR-512-3p. Additionally, the tumor-suppressive effect induced by circUBE2D2 depletion was greatly impaired upon miR512-3p down-regulation or CDCA3 overexpression. Also, depletion of circUBE2D2 decreased the resistance to doxorubicin through regulating miR-512-3p/CDCA3 axis.

**Conclusion:**

CircUBE2D2 promoted TNBC progression and doxorubicin resistance through acting as a sponge of miR-512-3p to up-regulate CDCA3 expression. Targeting circUBE2D2 combine with doxorubicin might be exploited as a novel therapy for TNBC.

## Background

Breast cancer ranks first in the newly reported cancer cases and cancer-related deaths among women [1]. As estimated, the incidence and mortality of female breast cancer account for 30% and 15% of the ten leading cancer types in the USA in 2019 [2]. Triple-negative breast cancer (TNBC) represents approximately 15–20% of all breast cancers that clinically are negative for the expression of estrogen receptor (ER), progesterone receptor (PR) and human epidermal growth factor receptor 2 (HER2) proteins [3]. Due to the aggressive behaviors, high risk of distant recurrence and lack of available targeted therapies, TNBC patients always carry a worse prognosis compared with other breast cancer subtypes [4]. Conventional chemotherapy remains the mainstay of treatment for TNBC, however, patients frequently develop resistance [5]. Thus, more efforts need to be put into understanding the molecular mechanisms involved in the initiation, progression and chemoresistance of TNBC in order to overcome the drug resistance and improve the clinical outcome.

Circular RNAs (CircRNAs) are a type of covalently closed single-stranded RNA molecules derived from the back-splicing of precursor mRNA with no 5′ cap structure and 3′-poly (A) tail [6]. During the past decades, circRNAs have been considered as the nonfunctional byproducts of splicing errors. With the booming development of high-throughput sequencing technology, circRNAs have been found to be abundantly expressed in eukaryotes with high stability and conservation at cell-, tissue-, or developmental stage-specific pattern [7]. A growing body of evidence demonstrates that circRNAs play vital roles in cellular physiology by serving as miRNA sponges, transcriptional regulators, RBP binding molecules, templates for protein translation, and immune regulators [8]. Increasing reports elucidate that circRNAs could act as tumor suppressors or promoters in kinds of human malignancies, highlighting their potential as diagnostic biomarkers and therapeutic targets in future personalized medicine [9, 10]. For instance, circOSBPL10 promoted cell growth and metastasis in gastric cancer by sponging miR-136-5p and up-regulating WNT2 [11]. Nonetheless, circHIPK3 retarded cell invasion, metastasis, and angiogenesis in bladder cancer via sponging miR-558 to suppress the expression of heparanase [12]. In recent years, a large number of circRNAs are revealed to be abnormally expressed in breast cancer, and participate in the carcinogenesis, metastasis or chemoresistance through different mechanisms [13, 14]. CircUBE2D2 (circBase ID: hsa_circ_0005728), generated by back-splicing of exon 2–5 of UBE2D2 mRNA, is located at chr5:138,979956–138,994,551 with a length of 280 bp. According to the circRNA expression profiles, hsa_circ_0005728 (circUBE2D2) expression was up-regulated in TNBC tissues compared with paracancerous tissues [15]. However, the biological functions of circUBE2D2 and its underlying molecular mechanisms are still obscure.

MicroRNAs (MiRNAs), conserved small non-coding RNAs with 18–25 nucleotides in length, are able to modulate various pathologic and physiological processes, such as cell cycle, differentiation, apoptosis, migration, inflammation, and stress response, through repressing translations and degrading mRNAs [16]. The correlation between miRNAs and cancer pathogenesis has been elucidated by a flood of literatures [17]. To our knowledge, circRNAs could serve as competing endogenous RNA (ceRNA) or miRNA sponges to prevent miRNAs from binding to their target genes [18]. Here, we made an attempt to explore whether circUBE2D2 was implicated in TNBC progression through the “circRNA-miRNA-mRNA” mode.

In the current study, we confirmed that circUBE2D2 expression was up-regulated in TNBC tissues and cells. High circUBE2D2 expression was positively correlated with TNM stage, lymph node metastasis and poor prognosis in TNBC patients. Functionally, silencing of circUBE2D2 inhibited cell proliferation, metastasis and doxorubicin resistance in vitro and impeded tumor growth in vivo. Mechanistically, circUBE2D2 could sponge for miR-512-3p to relieve its suppression on target cell division cycle associated 3 (CDCA3). In general, our findings provide a novel insight into the regulatory mechanism of circUBE2D2 and highlighted a potential therapeutic target in TNBC.

## Materials and methods

### TNBC tissue specimens

The 66 pairs of tumor tissues and paracancerous tissues were collected from patients who were diagnosed as TNBC by two experienced clinical pathologists at the First Affiliated Hospital of Zhengzhou University. No patients received local or systemic treatment before surgery. The excised fresh tissues were quickly immersed in liquid nitrogen and preserved at − 80 °C refrigerator.

### Cell culture

Human TNBC cells (BT-549, SUM-159, MDA-MB-231, MDA-MB-468, HCC38) and a normal mammary epithelial cell line MCF-10A were purchased from Shanghai Institute of Biological Sciences, Chinese Academy of Sciences (Shanghai, China). DMEM (Invitrogen, Carlsbad, CA, USA) containing 10% fetal bovine serum, 100 U/ml penicillin and 100 µg/ml streptomycin was used as the routine medium for cell culture. Cells were passaged for a period not exceeding 6 months. Cells were identified by short tandem repeat DNA profiling and confirmed no mycoplasma contamination.

### Plasmid construction and cell transfection

To down-regulate circUBE2D2, small interfering RNAs (siRNAs) targeting the back splice junction of circUBE2D2 (si-circUBE2D2#1, si-UBE2D2 #2) were synthesized by GenePharma (Shanghai, China). The non-specific oligonucleotide (si-NC) was used as negative control. To overexpress circUBE2D2, the circUBE2D2 cDNA was amplified and cloned into pcD-ciR vector (Geneseed, Guangzhou, China). To up-regulate CDCA3, the coding region of CDCA3 cDNA was inserted into pcDNA3.1 eukaryotic expression vector (Invitrogen, Carlsbad, CA, USA). To manipulate the expression of miR-512-3p, miR-512-3p mimics (miR-512-3p) or inhibitors (anti-miR-512-3p) were synthesized by GenePharma (Shanghai, China). TNBC cells were seeded in 6-well plates and cultured to reach approximately 60% confluence. Then, 50 nM oligonucleotides (including miRNA mimics/inhibitors or siRNAs) or 4 μg of plasmids were transfected into TNBC cells by using Lipofectamine 3000 (Invitrogen, Carlsbad, CA, USA) following the manufacturer’s instruction.

### Lentivirus packaging and cell transfection

The plasmids pLKO.1-sh-circUBE2D2 or corresponding negative control pLKO.1-sh-NC was purchased from GenePharma (Shanghai, China). To obtain lentiviruses, pLKO.1-sh-circUBE2D2 or pLKO.1-sh-NC were transfected into HEK293T cells together with the packaging plasmids psPAX2 and pVSVG according to the manufacturer’s guideline. After 48 h, virus supernatant was collected to infect MDA-MB-231 cells. Stable expressing cell lines were selected with puromycin (2 μg/ml) for 2 weeks.

### Quantitative real-time polymerase chain reaction (qRT-PCR)

TRIzol Reagent (Invitrogen) was used to exact the total RNA from frozen tissues and cultured cells. The nuclear and cytoplasmic portions of cellular RNAs were isolated by using PARIS Kit (Life Technologies, Austin, Texas, USA) according to the manufacturer’s guidelines. The complementary DNA (cDNA) was reversely transcribed by using PrimeScript RT Master Mix (TaKaRa, Japan). qRT-PCR reactions were performed on an ABI Prism 7500 sequence detection system (Applied Biosystems, Foster City, CA, USA) by using TB Green Premix Ex Taq (Takara, Dalian, China). GAPDH was used as a reference gene for circRNA and mRNA, and U6 was exploited as an internal control for miRNA. The 2^−ΔΔCt^ method was applied to quantify the relative expression of each gene. The primer sequences were listed in Table 1.

**Table 1:** The primer sequences used for qRT-PCR

Gene	Primer sequences (5′–3′)
circUBE2D2	Forward, GATCACAGTGGTCTCCAGCA
	Reverse, GCCCCATTATTGTAGCTTGC
UBE2D2	Forward, AGAATCCACAAGGAATTGAATGAT
	Reverse, TGGACAAGAGTACTTTTGAAATAG
miR-512-3p	Forward, AAGUGCUGUCAUAGCUGAGGUC
	Reverse, UUCUCCGAACGUGUCACGUTT
CDCA3	Forward, TGGTATTGCACGGACACCTA
	Reverse, TGTTTCACCAGTGGGCTTG
GAPDH	Forward, AGCCACATCGCTCAGACAC
	Reverse, GCCCAATACGACCAAATCC
U6	Forward, CTCGCTTCGGCAGCACA
	Reverse, AACGCTTCACGAATTTGCGT

### Confirmation of circUBE2D2 specificity

In order to validate the specificity of circUBE2D2, divergent and convergent primers were utilized to amplify the circular and linear transcripts of UBE2D2 in both cDNA and genomic DNA (gDNA) from TNBC cells. If a single band was obtained on agarose gel electrophoresis from the PCR products of circUBE2D2, then circUBE2D2 was deemed as specific.

### RNase R treatment

Total RNA (4 μg) isolated from TNBC cells was incubated for 30 min at 37 °C with or without 3U/μg RNase R (Epicenter Technologies, Madison, WI, USA). Then, qRT-PCR was performed to examine the expression levels of circUBE2D2 and UBE2D2 mRNA.

### Actinomycin D assay

TNBC cells were seeded into culture medium containing 2 mg/ml actinomycin D (Sigma-Aldrich, St. Louis, MO, USA). At 4, 8, 12, and 24 h, cells were harvested to detect the expression of circular and linear UBE2D2 by qRT-PCR analysis.

### Cell proliferation assay

Cell Counting Kit-8 (CCK-8; Beyotime, Shanghai, China) was applied to determine the cell viability. Transfected TNBC cells were inoculated into 96-well plates at a density of 2 × 10^3^ per well and routinely cultured under the standard environment. At 24, 48, 72 and 96 h, 10 μl CCK8 solution was added to each well for another 2 h of incubation. The absorbance was measured at 450 nm wavelength by using a microplate reader. Colony formation assay was used to detect the cell clonogenic ability. Transfected TNBC cells (500 cells/well) were seeded into 6-well plates and maintained at 37 °C for 12 days. Then, the cells were stained and the visible colonies were counted manually.

### Drug resistance assay

Transfected TNBC cells were inoculated into 96-well plates and treated with different concentrations of doxorubicin for 48 h. Then, the cell viability was determined using the CCK-8 method. Dose–response curves were drawn, from which the half-maximal inhibitory concentration (IC_50_) values of doxorubicin were determined with GraphPad Prism 7 (GraphPad Inc., San Diego, CA, USA). The IC_50_ values were used to evaluate the resistance of TNBC cells to doxorubicin.

### Transwell migration and invasion assay

TNBC cell migration and invasion were determined by using Costar Transwell 24-well plate (Costar Corning, Cambridge, MA, USA) pre-coated with (invasion assay) or without (migration assay) Matrigel. Briefly, 200 μl TNBC cell suspensions (5 × 10^4^ cells/well for migration, 1 × 10^5^/well for invasion) were added to the top chamber, and 600 μl medium with 5% FBS was placed into the lower chamber. After 24 h, cells adherent to the lower surface were stained, photographed and counted with a microscope.

### Apoptosis assay

Transfected TNBC cells were treated with or without indicated concentration of doxorubicin for 48 h. Then, flow cytometry analysis was performed to determine the apoptotic rate by using the Annexin V-FITC/PI Apoptosis Detection kit (Beijing Biosea Biotechnology, Beijing, China).

### Western blot assay

Total protein from TNBC cells was extracted by RIPA lysis buffer (Beyotime, Nantong, China) and quantified with a Pierce BCA Protein Assay Kit (Thermo Scientific, Rockford, IL, USA). Equal amount of protein (30 μg) was separated on 10% SDS-PAGE, transferred onto polyvinylidene fluoride (PVDF) membrane (Bio-Rad, Hercules, CA, USA), and then were probed with primary antibodies against Ki-67 (1:1000; ab243878, Abcam, Cambridge, MA, USA), CDCA3 (ab166902; 1:1000, Abcam), and GAPDH (1:5000, ab70699, Abcam) at 4 °C overnight. A HRP-conjugated secondary antibody was further used to incubate with the membranes at room temperature for 1 h. Finally, the protein bands were visualized by Immobilon ECL HRP substrate (Millipore, Billerica, MA, USA).

### Biotinylated RNA pull-down assay

The pull-down experiments with biotinylated-UBE2D2 probe or biotinylated-miR-512-3p were carried out as previously described by Li et al. [12]

### Luciferase reporter assay

The sequences of circUBE2D2 and CDCA3-3′UTR with (wild-type) or without (mutant) the miR-152-3p binding sites were synthesized and inserted into psiCHECK2 vector (Promega, Madison, WI, USA). TNBC cells (1 × 10^4^/well) were seeded in 96-well plates and cultured for 24 h. Then, cells were co-transfected with the established luciferase reporter (circUBE2D2-wt, circUBE2D2-mut, CDCA3-wt or CDCA3-mut) and miR-512-3p or miR-NC by using Lipofectamine 3000 (Invitrogen, Carlsbad, CA, USA). At 48 h post-transfection, Dual Luciferase Reporter Assay Kit (Promega, Madison, WI, USA) was used to determine the luciferase activity. Firefly luciferase was used as the internal reference to normalize the Renilla luciferase.

### RNA immunoprecipitation (RIP) assay

To identify the binding between circUBE2D2 and miR-512-3p, RIP assay was performed by using EZ-Magna RIP Kit (Millipore, Billerica, MA, USA) in TNBC cells after transfection with miR-512-3p or miR-NC. Briefly, cell lysates were incubated with RIP buffer containing magnetic beads conjugated with human anti-Ago2 antibody or control IgG antibody. After treatment with proteinase K, qRT-PCR was carried out to examine the enrichment of circUBE2D2 in the immunoprecipitated RNAs.

### Mice experiments

Five-week-old female BALB/c nude mice were kept in a specific pathogen-free environment and randomly allocated to two groups (n = 6 in each group). Constructed stable sh-NC- or sh-circUBE2D2-expressing MDA-MB-231 cells (5 × 10^6^) were subcutaneously inoculated into the right flanks of nude mice. The tumor volumes were determined following the formula (volume = width^2^ × length × 0.5). After 27 days, mice were killed by cervical dislocation and tumors were excised for further assays.

### Statistical analysis

All data were expressed as the mean ± standard deviation (SD) from three independent experiments. All statistical analyses were conducted and all figures were generated by using GraphPad Prism 7 (GraphPad Inc., San Diego, CA, USA). Two-tailed Student’s t-test or one-way analysis of variance (ANOVA) was used for the comparison of significance. Correlations were determined by using Pearson’s correlation coefficients. The cut-off value to stratify patients into high and low expression groups was defined as the median expression of objective gene. Kaplan–Meier method was used to create survival curves and log-rank test was applied to compare difference. All statistical tests were two-sided, and *P* < 0.05 denotes a statistical significance.

## Results

### CircUBE2D2 expression was up-regulated in TNBC and correlated to a poor prognosis

**Fig. 1:**
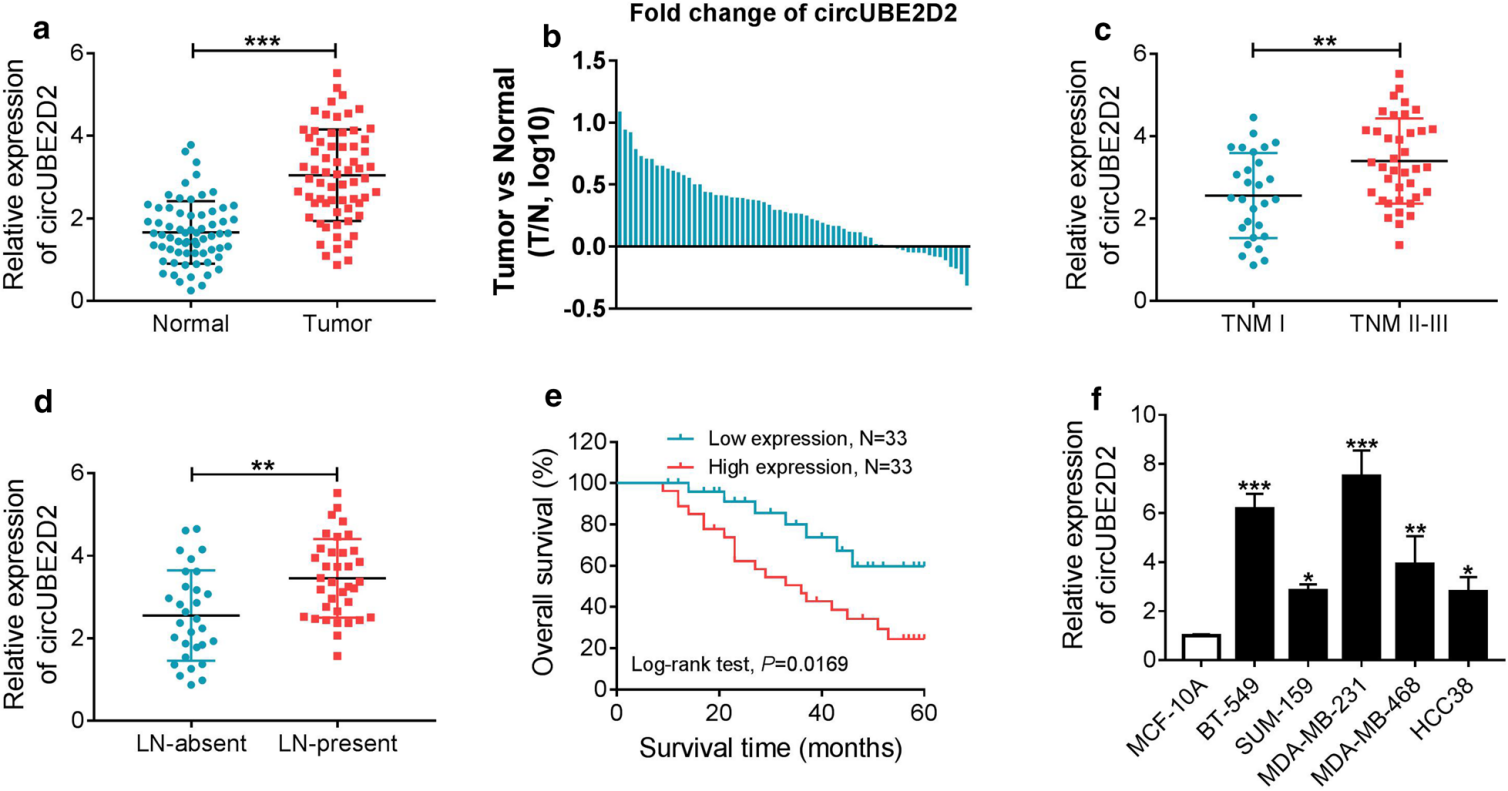
CircUBE2D2 is frequently increased and indicates a poor prognosis in TNBC. (**a** and **b**) Relative expression of circUBE2D2 in TNBC tissues (Tumor) and adjacent non-cancerous tissues (Normal) was determined by qRT-PCR. **c** Relative expression of circUBE2D2 in tumor tissues at different TNM stages. **d** Relative expression of circUBE2D2 in tumor tissues with or without lymph node metastasis. **e** Kaplan–Meier method was used to determine the correlation between circUBE2D2 expression and overall survival of TNBC patients. The median expression of circUBE2D2 was set as a cut-off value to divide patients into high expression group and low expression group. **f** Relative expression of circUBE2D2 in five TNBC cells and one normal mammary epithelial cell. *P < 0.05, **P < 0.01, ***P < 0.001

CircUBE2D2 expression was examined in 66 pairs of TNBC tumor tissues and neighboring non-cancerous tissues by qRT-PCR. The result showed that circUBE2D2 expression was higher in cancer tissues than that in corresponding normal tissues (Fig. 1a). Among these paired specimens, circUBE2D2-overexpressing tumor (n = 55) tissues took up about 75% (Fig. 1b). Moreover, increased circUBE2D2 expression was observed in tumor tissues at TNM II-III stage compared to that at TNM I stage (Fig. 1c). CircUBE2D2 expression was up-regulated in tumor tissues with lymph node metastasis compared with that without lymph node metastasis (Fig. 1d). Kaplan–Meier survival analysis revealed a poorer overall survival in TNBC patients with high circUBE2D2 expression (Fig. 1e). In accordant with the expression status in tumor tissues, the level of circUBE2D2 in TNBC cell lines was elevated when compared to normal mammary epithelial cell line MCF-10A, particularly in MDA-MB-231 and BT-549 cells (Fig. 1f). Collectively, abnormal expression of circUBE2D2 might be correlated to TNBC progression and prognosis.

### Validation of circular structure of circUBE2D2

**Fig. 2:**
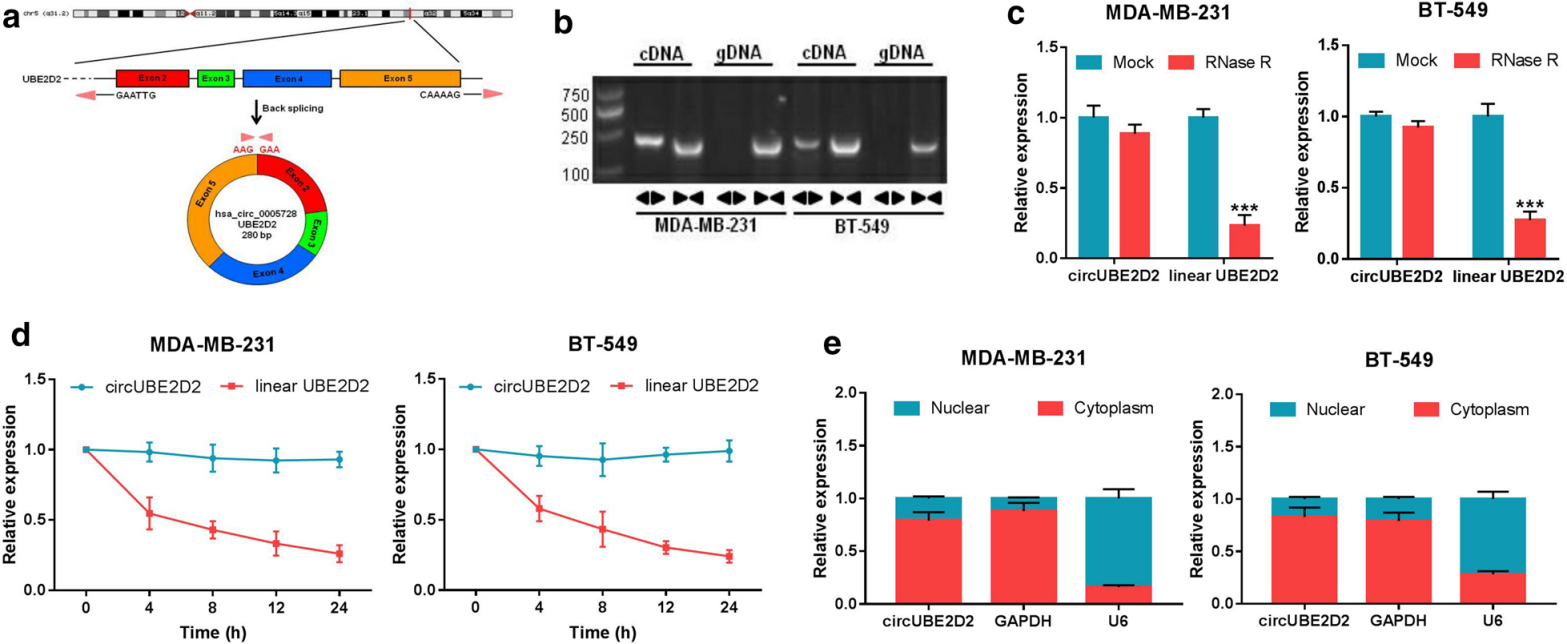
Characterization of circUBE2D2 in TNBC cells. **a** CircUBE2D2 is located at chromosome 5q31.2 and generated by via the circularization of exon 2–5 in UBE2D2 gene. **b** Divergent and convergent primers were used to amplify the circular and linear UBE2D2 with cDNA or gDNA as the template in MDA-MB-231 and BT-549 cells. **c** Relative expression of circUBE2D2 and UBE2D2 mRNA in MDA-MB-231 and BT-549 cells after treatment with RNase R. **d** Relative expression of circUBE2D2 and UBE2D2 mRNA at different time points in MDA-MB-231 and BT-549 cells after treatment with actinomycin D. **e** Nuclear-cytoplasmic fractionation assay was performed to examine the subcellular distribution of circUBE2D2 in MDA-MB-231 and BT-549 cells. ***P < 0.001

According to the data from USCS and circBase, circUBE2D2 (hsa_circ_0005728) is derived from exon 2–5 of UBE2D2 gene located on chromosome 5q31.2 by back-splicing and is 280 bp in length (Fig. 2a). Subsequently, the divergent and convergent primers were designed to amplify the circular transcripts and linear transcripts of UBE2D2, respectively. By using cDNA and gDNA from MDA-MB-231 and BT-549 cells as templates, gel electrophoresis results manifested that circUBE2D2 was only amplified by divergent primers in cDNA, while no amplification product was observed in gDNA (Fig. 2b). Also, it was found that the linear UBE2D2 was degraded by RNase R treatment, but circUBE2D2 was resistant to RNase R digestion (Fig. 2c). Additionally, actinomycin D (an inhibitor of transcription) treatment led to a significant suppression of linear UBE2D2 expression rather than circUBE2D2 expression (Fig. 2d). According to the data of subcellular fractionation, circUBE2D2 was predominantly located in the cytoplasm of MDA-MB-231 and BT-549 cells (Fig. 2e). All these data verified circUBE2D2 as a circular structure.

### Silencing of circUBE2D2 inhibited cell proliferation, migration and invasion in TNBC

**Fig. 3:**
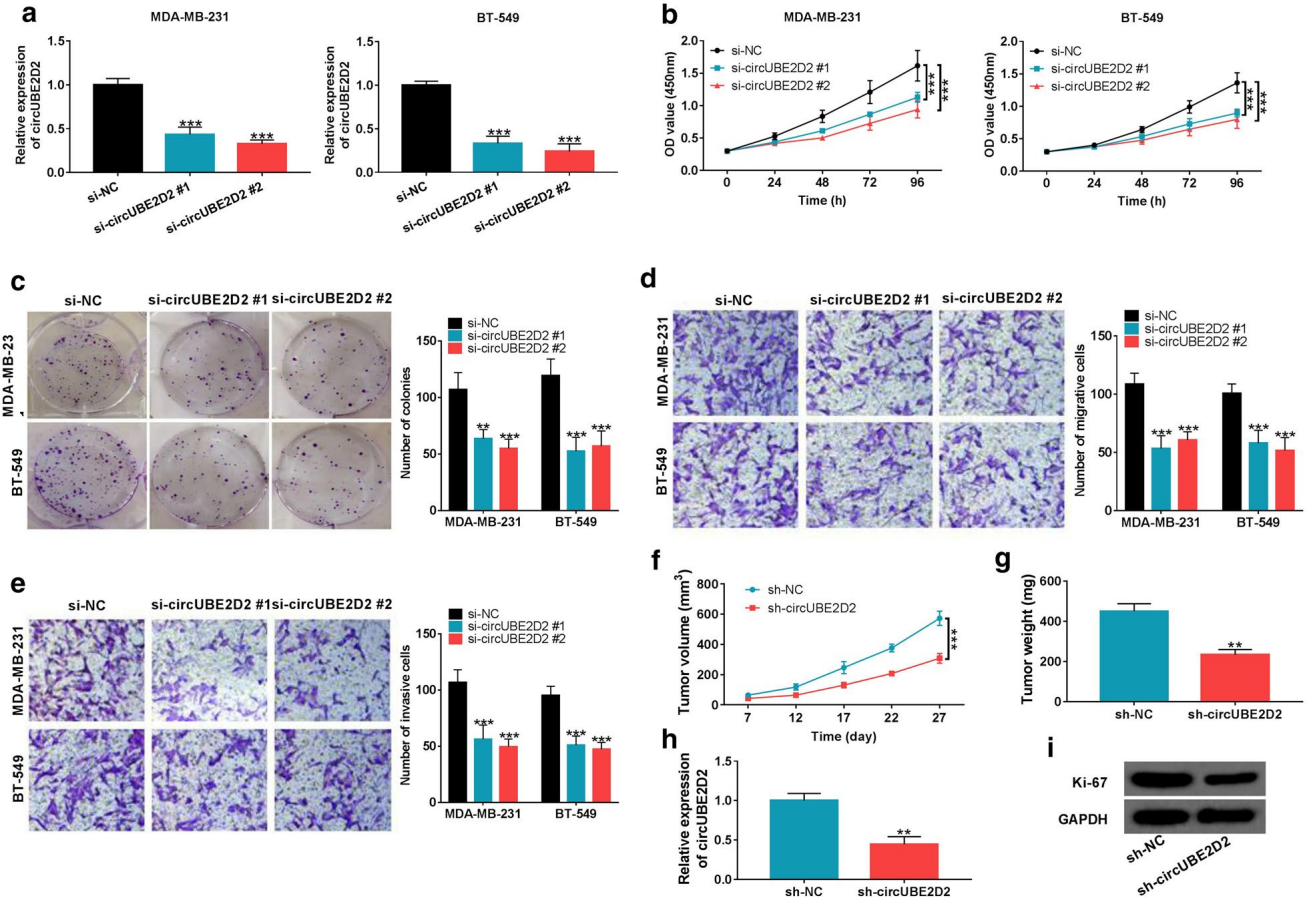
Knockdown of circUBE2D2 inhibits TNBC progression in vitro and in vivo. **a** The interfering efficiency of two siRNAs targeting circUBE2D2 (circUBE2D2 #1 and circUBE2D2 #2) were examined by qRT-PCR. **b** and **c** CCK-8 and colony formation assays were conducted to determine the influence of circUBE2D2 silencing on TNBC cell proliferation. **d** and **e** Cell migration and invasion were detected by transwell assay in MDA-MB-231 and BT-549 cells transfected with si-circUBE2D2 #1 or si-circUBE2D2 #2. **f** Xenografts growth curves during the time course of 27 days. **g** Tumor weights were monitored after the nude mice were euthanized. **h** CircUBE2D2 expression analysis in the resected tumor tissues. **i** Western blot analysis of Ki-67 protein in tumors excised from mice. **P < 0.01, ***P < 0.001

To probe into the biological functions of circUBE2D2 in TNBC, two different siRNAs targeting the junction sites of circUBE2D2 (si-circUBE2D2 #1, si-circUBE2D2 #2) were transfected into MDA-MB-231 and BT-549 cells. As shown in Fig. 3a, circUBE2D2 expression was effectively suppressed. CCK-8 and colony forming assays were performed to determine the influence of circUBE2D2 on cell proliferation. Knockdown of circUBE2D2 significantly repressed TNBC cell growth (Fig. 3b). Accordingly, the colony forming ability of MDA-MB-231 and BT-549 cells were both attenuated upon the down-regulation of circUBE2D2 (Fig. 3c). Transwell assays were used to measure the effect of circUBE2D2 on cell metastatic capability. The result manifested that cell migration and invasion were obviously abated with the decrease of circUBE2D2 expression (Fig. 3d and e). Furthermore, xenograft mouse models were built to investigate the role of circUBE2D2 in TNBC in vivo. The tumor volumes and weights were evidently lowered in circUBE2D2-knockdown group (Fig. 3f and g). As expected, circUBE2D2 expression was reduced in tumors developed from sh-circUBE2D2-transfected cells compared to sh-NC group (Fig. 3h). And, the protein expression of proliferation marker Ki-67 in excised tumors was inhibited by the depletion of circUBE2D2 (Fig. 3i). To sum up, circUBE2D2 exerted an oncogenic property in TNBC in vitro and in vivo.

### Knockdown of circUBE2D2 decreased the chemoresistance of TNBC cells to doxorubicin

**Fig. 4:**
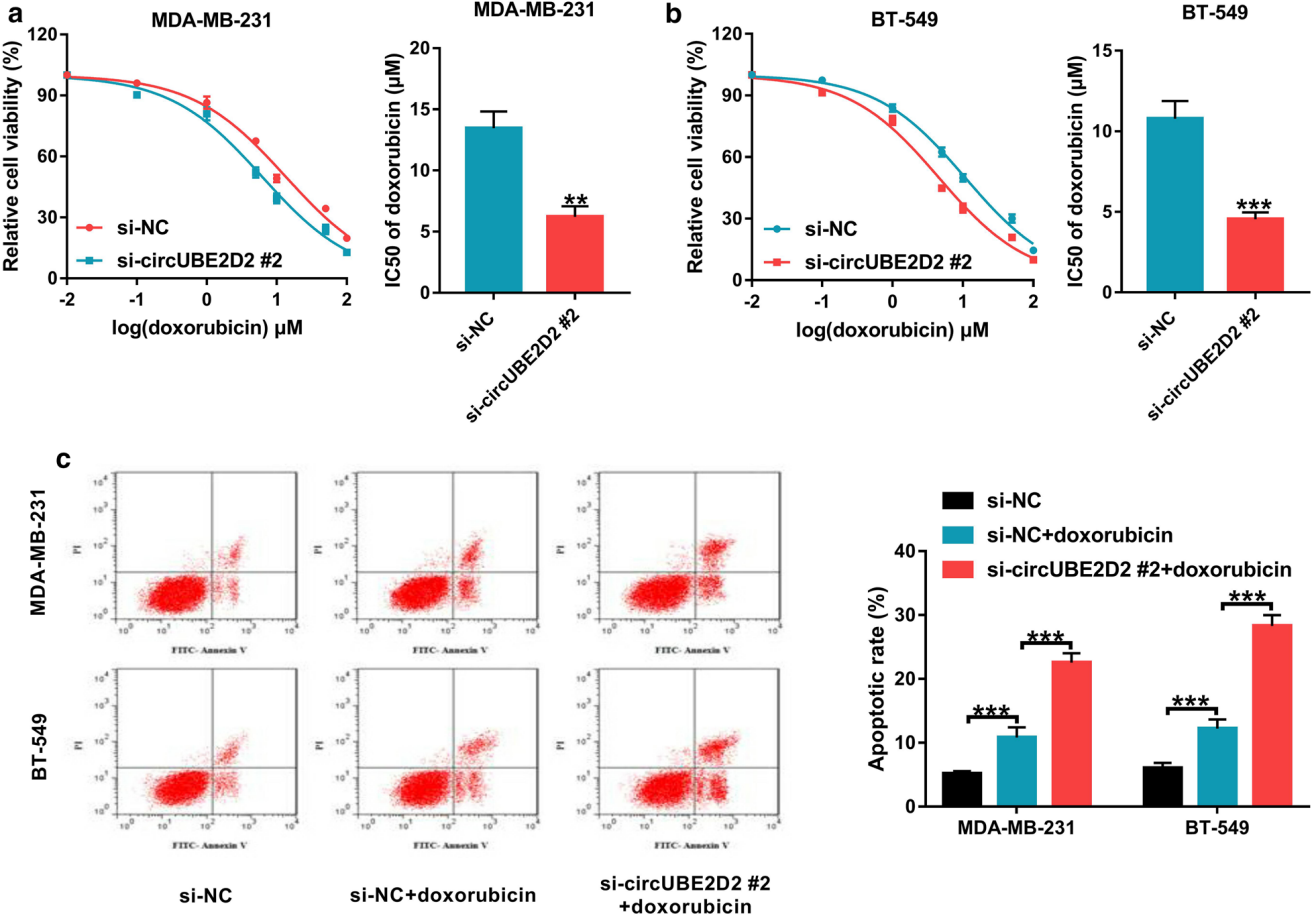
Knockdown of circUBE2D2 reduces doxorubicin resistance in TNBC cells. **a** and **b** Transfected MDA-MB-231 and BT-549 cells were treated with different concentrations of doxorubicin (0.01, 0.1, 1, 5, 10, 50 or 100 μM) for 48 h, followed by CCK-8 assay to measure the cell viability and IC_50_ values of doxorubicin. **c** Transfected MDA-MB-231 and BT-549 cells were treated with or without 2 μM doxorubicin for 48 h, followed by flow cytometry analysis to detect cell apoptosis. **P < 0.01, ***P < 0.001

Doxorubicin-based chemotherapy is the most frequently used treatment for TNBC [19]. Here, we investigated the impact of circUBE2D2 on doxorubicin resistance in TNBC cells. As presented in Fig. 4a and b, depletion of circUBE2D2 decreased the cell viability and IC_50_ values as compared with those in si-NC-transfected cells. Moreover, silencing of circUBE2D2 enhanced doxorubicin-induced apoptosis (Fig. 4c). Together, knockdown of circUBE2D2 could reduce doxorubicin resistance of TNBC cells.

### CircUBE2D2 acted as a molecular sponge for miR-512-3p

**Fig. 5:**
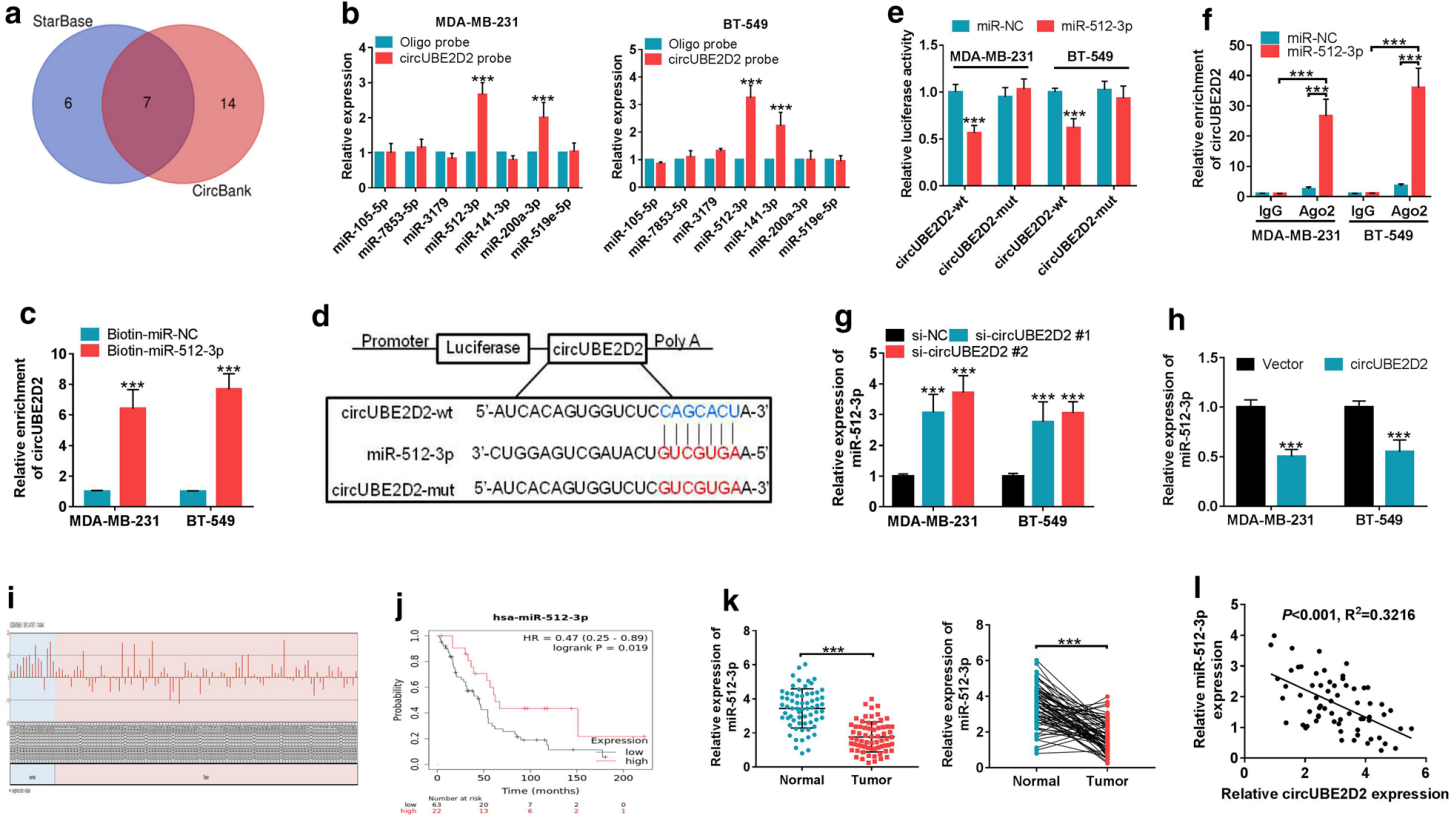
CircUBE2D2 serves as a sponge for miR-512-3p in TNBC cells. **a** CircBank and StarBase 3.0 predicted seven miRNAs containing the potential binding sites of circUBE2D2. **b** Biotinylated-circUBE2D2 pull-down assay was performed in MDA-MB-231 and BT-549 cells, followed by qRT-PCR to measure the enrichment of seven candidate miRNAs. **c** Biotinylated-miR-512-3p and control Biotinylated-miR-NC were transfected into MDA-MB-231 and BT-549 cells with circUBE2D2 overexpression, and qRT-PCR was used to test the level of circUBE2D2 in qRT-PCR immunoprecipitates. **d** Schematic diagram of wild-type or mutant circUBE2D2 luciferase reporter vectors. **e** Effects of miR-512-3p overexpression on the luciferase activity of MDA-MB-231 and BT-549 cells transfected with wild-type or mutant circUBE2D2 luciferase reporter vector. **f** Anti-Ago2 RIP was carried out in MDA-MB-231 and BT-549 cells transfected with miR-512-3p or miR-NC, followed by qRT-PCR to measure the level of circUBE2D2. **g** and **h** qRT-PCR was utilized to evaluate the effects of circUBE2D2 silencing or overexpression on miR-512-3p expression. **I** The expression difference of miR-512-3p in breast cancer tissues and normal tissues was displayed according to the data from GSE45666. **j** Kaplan–Meier analysis for the correlation between miR-512-3p expression and overall survival of breast cancer patients from GSE40267 data. **k** qRT-PCR analysis of miR-512-3p expression in tumor tissues and corresponding para-cancerous tissues. **l** Pearson’s correlation coefficients were applied to assess the association of miR-512-3p and circUBE2D2 in TNBC tissues. ***P < 0.001

It is acknowledged that serving as miRNA sponges is the most common action mechanism for circRNAs in the cytoplasm. To investigate whether circUBE2D2 exerted the carcinogenic role through the similar pattern, StarBase 3.0 and CircBank databases were used to predict the miRNAs that could potentially interact with circUBE2D2. As displayed in Fig. 5a, the overlapping portions in Venn diagram displayed seven candidate miRNAs (hsa-miR-105-5p, hsa-miR-7853-5p, hsa-miR-3179, hsa-miR-512-3p, hsa-miR-141-3p, hsa-miR-200a-3p, hsa-miR-519e-5p). Among these 7 miRNAs, miR-512-3p was the only one that could be captured by circUBE2D2 probe in both MDA-MB-231 and BT-549 cells (Fig. 5b). Furthermore, RNA pull-down experiments with biotinylated miRNAs uncovered that more circUBE2D2 was caught by biotin-labeled miR-512-3p compared with control group in MDA-MB-231 and BT-549 cells with circUBE2D2 overexpression (Fig. 5c). The wild-type sequences of circUBE2D2 possessing the miR-512-3p binding sites and its mutant version in which the miR-512-3p binding sites were impaired were inserted into the luciferase reporter vector psiCHECK2 (Fig. 5d). Dual-luciferase reporter assay results showed that compared with miR-NC group, overexpression of miR-512-3p reduced the luciferase activity of wild reporter by nearly 40%, but made no difference to the luciferase activity of circUBE2D2-mut reporter (Fig. 5e). RIP assay delineated that compared with miR-NC group, circUBE2D2 was greatly enriched by miR-512-3p in Ago2 antibody (Fig. 5f). Moreover, miR-512-3p expression was promoted by the knockdown of circUBE2D2, while was inhibited upon the overexpression of circUBE2D2 in TNBC cells (Fig. 5g and h). Data from GSE45666 demonstrated a down-regulation of miR-512-3p expression in breast cancer tissues in contrast to normal tissues (Fig. 5i). Additionally, breast cancer patients with high miR-512-3p expression carried a better prognosis according to GSE40267 data (Fig. 5j). Here, a significant drop of miR-512-3p expression was also observed in TNBC tumor samples compared to that in paraneoplastic tissues (Fig. 5k). It is worth noting that miR-512-3p expression was negatively correlated with circUBE2D2 expression in TNBC tissues (Fig. 4l). From the above, circUBE2D2 could function as an endogenous sponge for miR-512-3p in TNBC cells.

### CircUBE2D2 up-regulated CDCA3 expression through sponging miR-512-3p

**Fig. 6:**
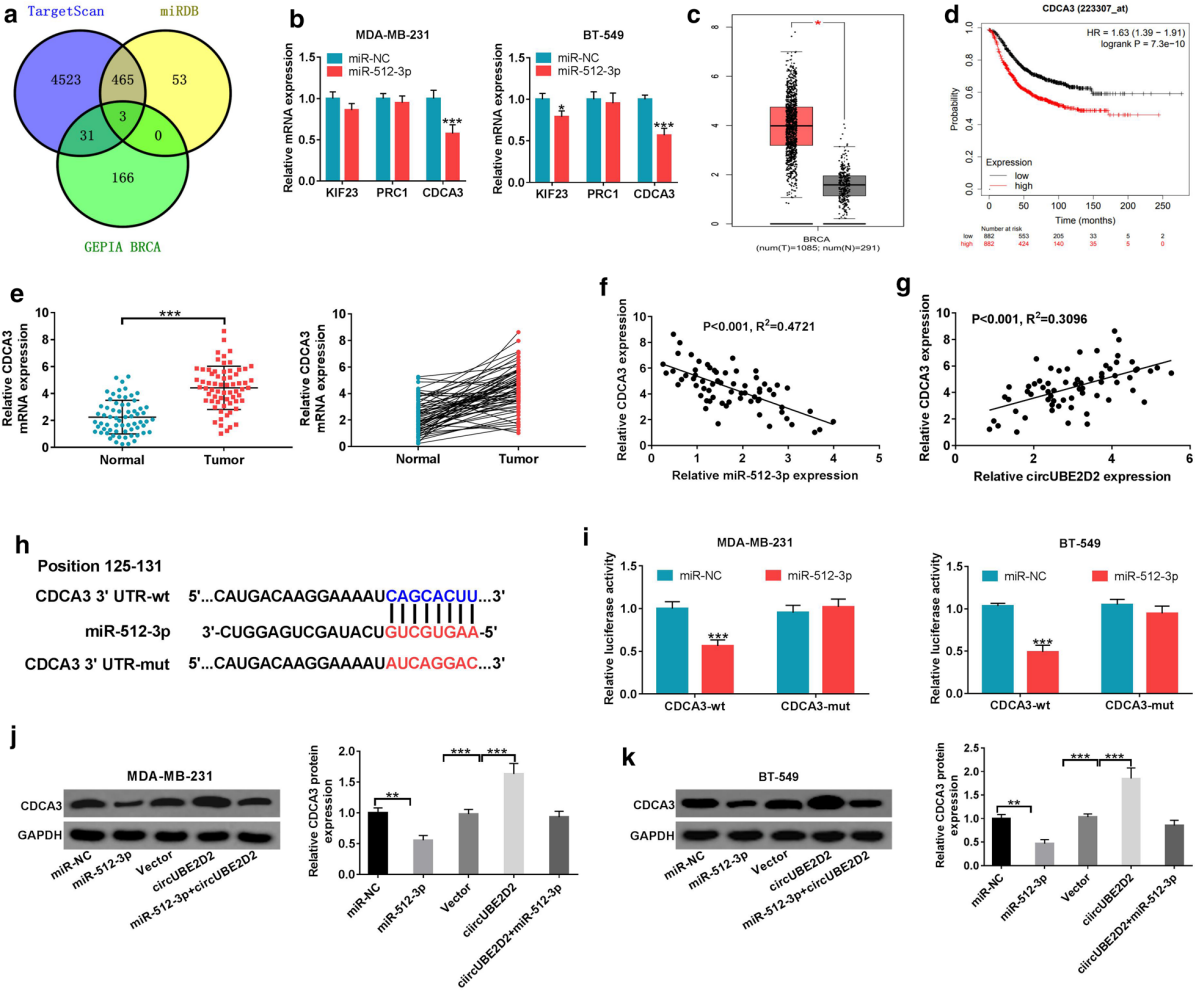
CircUBE2D2 sponges miR-512-3p to relieve its suppression on target gene CDCA3 in TNBC cells. **a** The potential targets of miR-512-3p predicted by TargetScan and miRTarBase were narrowed down to three by comparison with the top 200 up-regulated genes in breast cancer from GEPIA. **b** The effect of miR-512-3p on the expression of three candidate genes (KIF23, PRC1, CDCA3) was determined by qRT-PCR. **c** GEPIA database displayed the expression of CDCA3 mRNA in breast cancer tissues and normal tissues. **d** Kaplan–Meier analysis uncovered the correlation between CDCA3 expression and overall survival of breast cancer patients. **e** The expression level of CDCA3 mRNA in tumor tissues and proximal normal tissues from 66 TNBC patients was examined through qRT-PCR. **f** and **g** Pearson test was used to analyze the correlation between CDCA3 and miR-512-3p or circUBE2D2 in TNBC tumor tissues. **h** Putative complementary sites of miR-512-3p on the CDCA3-3′UTR. **i** The dual-luciferase activity was measured in MDA-MB-231 and BT-549 cells after co-transfected wild-type or mutant CDCA3 reporter and miR-512-3p or miR-NC. **j** and **k** Western blot assay was conducted to detect the effect of miR-512-3p or circUBE2D2 on CDCA3 protein level. **P < 0.01, ***P < 0.001

With the assistance of bioinformatics softwares TargetScan and miRTarBase, 468 possible targets of miR-512-3p were predicted. By comparison with the top 200 up-regulated genes in breast invasive carcinoma (BRCA) from GEPIA database, only three genes (KIF23, PRC1, CDCA3) were screened for further research (Fig. 6a). Next, qRT-PCR was performed to evaluate the influence of miR-512-3p on the expression of these three genes. As a result, CDCA3 was found to be effectively suppressed by miR-512-3p overexpression in MDA-MB-231 and BT-549 cells (Fig. 6b). According to the data from TCGA, CDCA3 expression was up-regulated and associated with a worse prognosis in breast cancer (Fig. 6c and d). In fact, higher CDCA3 mRNA expression was verified in 66 TNBC cancerous tissues than that in matched normal tissues (Fig. 6e). Additionally, CDCA3 expression was inversely associated with miR-512-3p expression, while was positively correlated with circUBE2D2 expression in TNBC tumor tissues (Fig. 6f and g). The complementary base pairs between CDCA3-3′UTR and miR-512-3p were depicted in Fig. 6h. Dual-luciferase reporter assay disclosed that enforced expression of miR-512-3p significantly reduced the luciferase activity of wide type CDCA3 reporter, but this effect was disappeared when these binding sites were mutated (Fig. 6i). Furthermore, the protein level of CDCA3 was decreased by miR-512-3p overexpression, while was enhanced upon circUBE2D2 up-regulation. Moreover, circUBE2D2-induced increase of CDCA3 protein expression was reversed when miR-512-3p was co-transfected into cells (Fig. 6j and k). These aforementioned results supported this conclusion that circUBE2D2 sponged miR-512-3p to attenuated its suppression on CDCA3 expression.

### Tumor-suppressive effects induced by circUBE2D2 knockdown were abated by miR-512-3p down-regulation or CDCA3 overexpression in TNBC

**Fig. 7:**
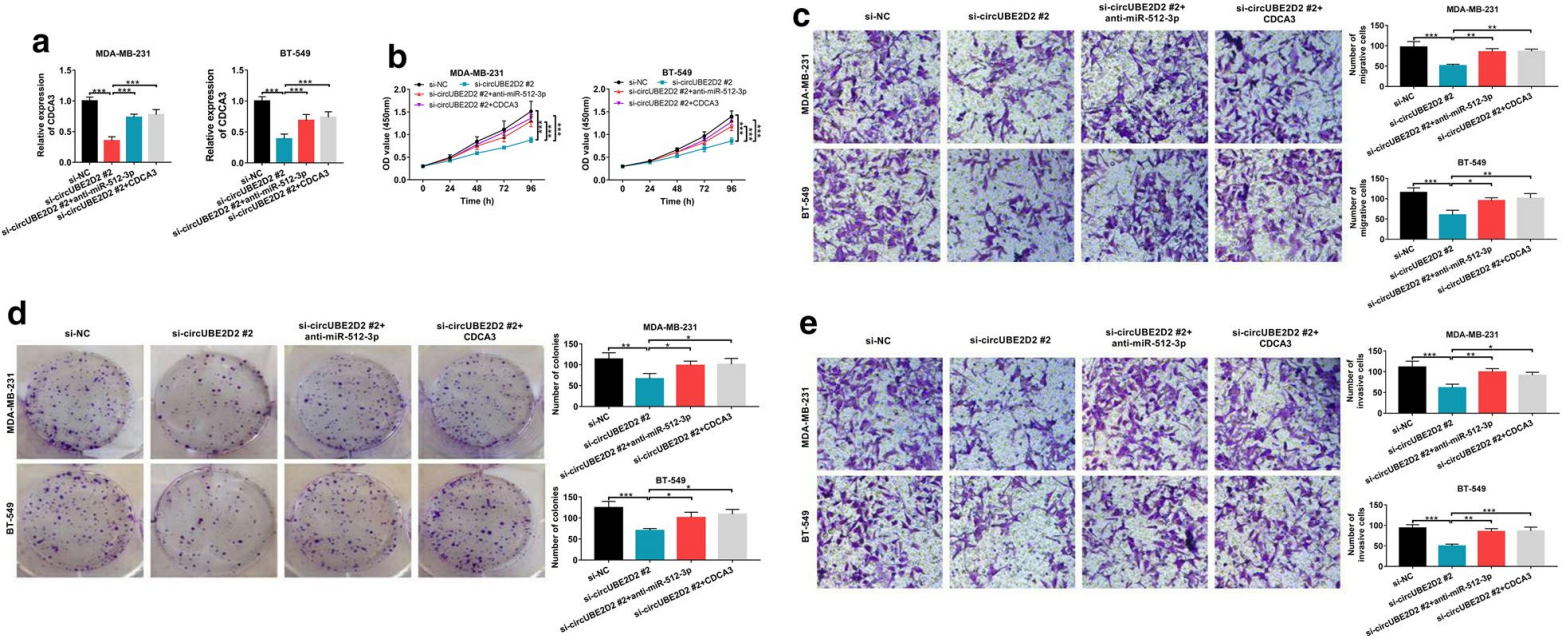
Down-regulation of miR-512-3p or overexpression of CDCA3 antagonizes the anti-cancer effects induced by circUBE2D2 knockdown in TNBC cells. **a**–**e** MiR-512-3p inhibitor (anti-miR-512-3p) or CDCA3-overexpression plasmid (CDCA3) was transfected into MDA-MB-231 and BT-549 cells with circUBE2D2 knockdown. **a** The expression level of miR-512-3p was measured by qRT-PCR. **b** and **c** The cell proliferation ability was examined by CCK-8 and colony formation assays. **d** and **e** The cell migration and invasion were evaluated by transwell assay. *P < 0.05, **P < 0.01, ***P < 0.001

To figure out whether circUBE2D2 promoted TNBC development via regulating miR-512-3p/CDCA3 pathway, circUBE2D2-knockdown MDA-MB-231 and BT-549 cells were transfected with anti-miR-512-3p or pcDNA-CDCA3. As exhibited in Fig. 7a, si-circUBE2D2-induced decrease of CDCA3 expression was partially rescued by the suppression of miR-512-3p or overexpression of CDCA3. Likewise, circUBE2D2 silencing mediated suppression effects on cell proliferation (Fig. 7b), colony formation (Fig. 7c), migration (Fig. 7d) and invasion (Fig. 7e) were greatly abrogated following the depletion of miR-512-3p or up-regulation of CDCA3. Above all, circUBE2D2 contributed to cell malignant phenotypes in TNBC by regulating miR-512-3p/CDCA3 axis.

### Knockdown of circUBE2D2 reduced doxorubicin resistance of TNBC cells by regulating miR-512-3p/CDCA3 axis

**Fig. 8:**
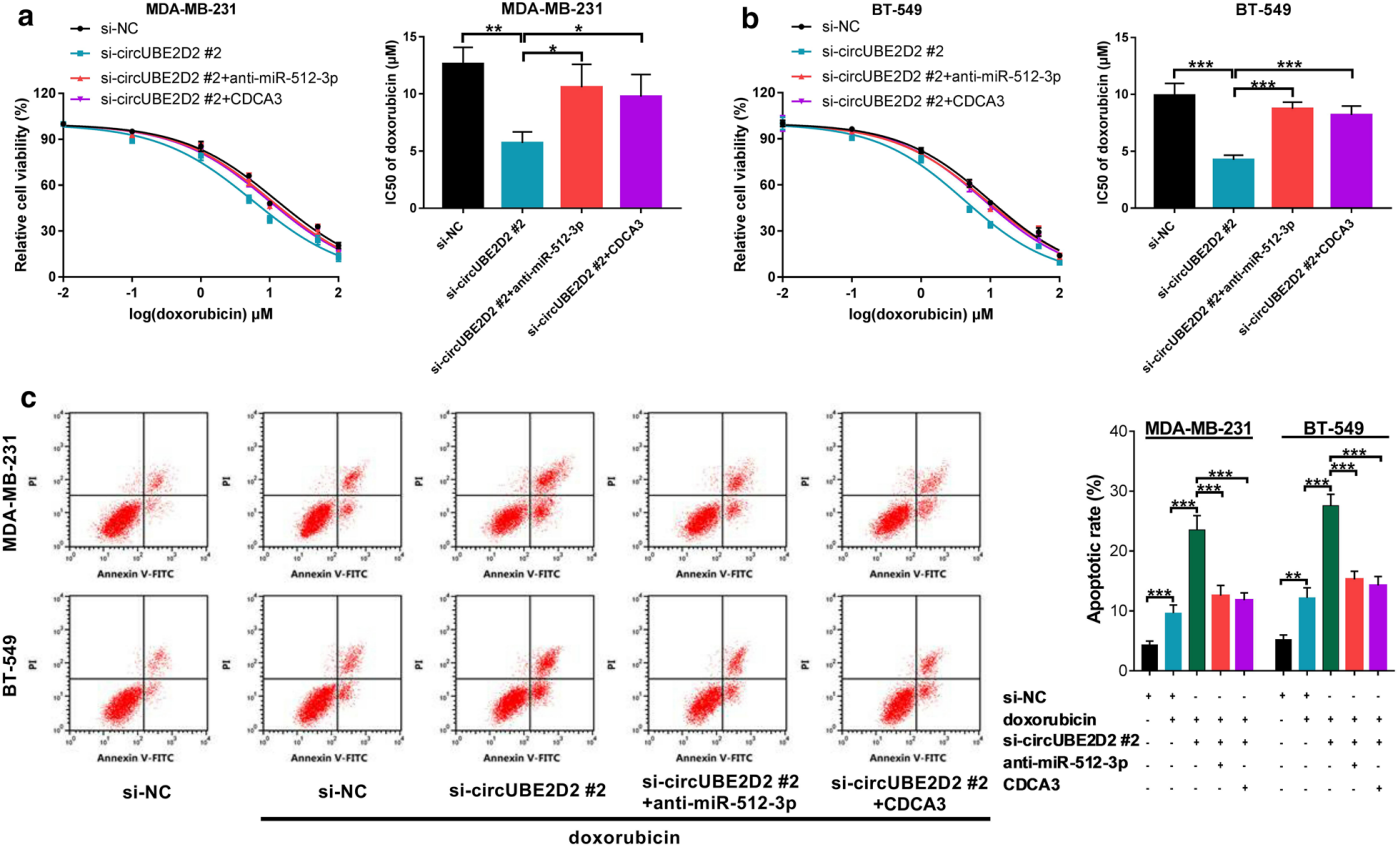
CircUBE2D2 induced doxorubicin resistance in TNBC cells through regulating miR-512-3p/CDCA3. **a** and **b** Transfected MDA-MB-231 and BT-549 cells were treated with different doses of doxorubicin (0.01, 0.1, 1, 5, 10, 50 or 100 μM) for 48 h, and the cell viability and IC_50_ values of doxorubicin were then determined using a CCK-8 assay. **c** Transfected MDA-MB-231 and BT-549 cells were treated with or without 2 μM doxorubicin for 48 h, and cell apoptosis was then assessed by flow cytometry. **P < 0.01, ***P < 0.001

Subsequently, we further addressed whether circUBE2D2 could confer doxorubicin resistance through modulating miR-512-3p/CDCA3. The rescue experiment results showed that si-circUBE2D2-mediated loss of doxorubicin resistance was substantially reversed by miR-512-3p down-regulation or CDCA3 overexpression (Fig. 8a and b). Moreover, si-circUBE2D2-induced apoptosis in TNBC cells treated with doxorubicin was dramatically eliminated due to the inhibition of miR-512-3p or overexpression of CDCA3 (Fig. 8c). Thus, it was concluded that circUBE2D2 enhanced doxorubicin resistance in TNBC cells by regulating miR-512-3p/CDCA3 axis.

## Discussion

Owing to the aggressive nature and absence of targeted therapies, TNBC patients always present an inferior prognosis [20]. CircRNAs have recently become a hot research topic for their crucial involvement in oncogenesis and cancer progression [21]. CircRNAs are considered as more appropriate tumor biomarkers than other RNAs duo to their high abundance and stability. Up to now, many circRNAs are still not well characterized in biological functions and molecular mechanisms. The present study demonstrated that circUBE2D2 exerted an oncogenic role and induced doxorubicin resistance in TNBC through sponging miR-512-3p to up-regulate CDCA3 expression.

A growing number of circRNAs have been elucidated as oncogenes or tumor-suppressors in the development of breast cancer. For instance, circRAD18 facilitated cell growth and metastasis in TNBC via sponging miR-208a/3164 to affect IGF1 and FGF2 expression [22]. CircKDM4C repressed tumor progression and decreased doxorubicin resistance by modulating miR-548p/PBLD axis in breast cancer [23]. In the current study, we found that circUBE2D2 expression was up-regulated in TNBC, and associated with TNM stage, lymph node metastasis and poor prognosis. CircUBE2D2 was validated as circular structure by PCR analysis, RNase R and actinomycin D treatment. Loss of function experiments showed that silencing of circUBE2D2 suppressed cell proliferation, migration, invasion and doxorubicin resistance in vitro, and slowed tumor growth in vitro. That is to say, circUBE2D2 served as a cancerogenic factor in TNBC. Consistent with our finding, a recent document reported that circUBE2D2 promotes breast cancer progression by sponging miR-1236 and miR-1287 [24].

With multiple miRNA-binding sites or miRNA response elements, circRNAs have been accepted as “miRNA sponges” to regulate their expression and activity [25]. CircRNAs derived from exons typically exist in the cytoplasm and may act as miRNA sponges [26]. Here, circUBE2D2 was found to be predominantly localized in the cytoplasm. Thus, we presumed that circUBE2D2 participated in the regulation of TNBC depending on this model of action. According to the information from bioinformatic algorithms StarBase and CircBank, seven miRNAs were selected as candidate targets of circUBE2D2. Through a series of bioinformatics analysis, miR-512-3p was revealed as a direct target of circUBE2D2. MiR-512-3p was proved as a tumor-suppressor in several human tumors, such as non-small cell lung cancer (NSCLC) [27] and hepatocellular carcinoma [28]. A recent literature clarified that overexpression of miR-512-3p inhibited malignant tumor behavior and drug resistance in breast cancer by targeting Livin [29]. In the current research, miR-512-3p expression was confirmed be decreased in TNBC tissues, and negatively associated with circUBE2D2 expression. Thereby, it was concluded that circUBE2D2 contributed to the malignant phenotypes of TNBC by sponging miR-512-3p.

Subsequently, CDCA3 was demonstrated as a target of miR-512-3p by using bioinformatics analysis and luciferase reporter assay. CDCA3, a trigger of mitotic entry, is frequently highly expressed and recognized as an oncogene in a variety of human malignancies. For instance, knockdown of CDCA3 inhibited cellular proliferation by arresting cell-cycle progression at the G1 phase in oral cancer [30]. Suppression of CDCA3 decreased cell proliferative, healing, or invasive ability in gastric cancer [31, 32]. Also, CDCA3 was reported to exert a carcinogenic effect in NSCLC and colorectal cancer via different signaling pathway [33–35]. In breast cancer, CDCA3 expression was found to be up-regulated and correlated with an unfavorable prognosis [36]. The collective evidence suggested that CDCA3 exerted tumor-promoting action in breast cancer. Similarly, we observed an up-regulation of CDCA3 expression in TNBC tissues. Besides, CDCA3 expression was positively correlated with circUBE2D2, while was negatively associated with miR-512-3p in TNBC tissues. Furthermore, CDCA3 expression was inhibited by miR-512-3p, while was elevated by circUBE2D2 in MDA-MB-231 and BT-549 cells. CircUBE2D2-mediated increase of CDCA3 was reversed following co-transfection with miR-512-3p. What’s more, si-circUBE2D2-induced suppressive effect on cell proliferation and metastasis was obviously abolished under the down-regulation of miR-512-3p or overexpression of CDCA3. The generation and development of drug resistance are an enormous challenge to the chemotherapy in TNBC patients [37]. In this study, we found that knockdown of circUBE2D2 reduced the doxorubicin resistance through facilitating doxorubicin-induced apoptosis via moludating miR-512-3p and CDCA3. Likewise, a recent document showed that CDCA3 expression was increased in TNBC and associated with a poor prognosis, and depletion of CDCA3 enhanced sensitivity to chemotherapy [38]. On the whole, circUBE2D2 served as a sponge to protect CDCA3 from being attacked by miR-512-3p, leading to TNBC progression and doxorubicin resistance. Nevertheless, further investigations are warranted due to the complicated regulatory networks of circRNAs in cancer malignancy. Apart from serving as “miRNA sponges”, circRNAs are also able to act as protein scaffolds, regulators of transcription and splicing, as well as occasional templates for polypeptide production [39]. In the subsequent research, we will further uncover the other potential regulation mechanisms for circUBE2D2 in TNBC.

## Conclusion

In general, circUBE2D2 was highly expressed in TNBC tissues and cells. Increased circUBE2D2 expression was associated with TNM stage, lymph node metastasis and poor prognosis. Functionally, knockdown of circUBE2D2 suppressed cell proliferation, migration, invasion and doxorubicin resistance. Mechanistically, circUBE2D2 acted as a sponge for miR-512-3p to modulate the expression of CDCA3. These observations help us to gain a further understanding of circRNA action mechanisms. Our findings also highlight a promising approach for combination chemotherapy of doxorubicin with targeting circRNAs in TNBC.

## Data Availability

The data sets used and/or analyzed during the current study are available from the corresponding author on reasonable request.

## Abbreviations

TNBC: Triple-negative breast cancer
circRNAs: Circular RNAs
CDCA3: Cell division cycle associated 3
ER: Estrogen receptor
PR: Progesterone receptor
HER2: Human epidermal growth factor receptor 2
ceRNA: Competing endogenous RNA
cDNA: Complementary DNA
gDNA: Genomic DNA
CCK-8: Cell Counting Kit-8
IC50: Half-maximal inhibitory concentration
RIP: RNA immunoprecipitation
NSCLC: Non-small cell lung cancer
